# High‐Frequency Ultrasonographic Characterization of Subcutaneous Glomus Tumors

**DOI:** 10.1002/jcu.70153

**Published:** 2025-12-03

**Authors:** Mingyu Bai, Nan Wang, Jie Jiang

**Affiliations:** ^1^ Department of Ultrasound Peking University Third Hospital Beijing China; ^2^ Department of Gynaecology and Obstetrics Peking University Third Hospital Beijing China

**Keywords:** glomus tumor, high‐frequency ultrasound, osseous manifestations, vascular stalk sign

## Abstract

**Objective:**

To evaluate the clinical manifestations and ultrasonographic characteristics of glomus tumors under the nails and other subcutaneous soft tissues.

**Methods:**

A retrospective analysis was performed on 45 patients with pathologically confirmed glomus tumors who underwent ultrasonographic examination at our institution between October 15, 2019, and April 8, 2025. Twenty tumors were in subungual and 25 were in extrasubungual subcutaneous tissue. Clinical parameters and ultrasonographic findings by a linear transducer (15–18 MHz) were systematically documented.

**Results:**

Among the 45 glomus tumors, the maximum tumor diameter ranged from 0.2 to 2.8 cm. Ultrasonographic features showed that 93.3% of lesions were hypoechoic, 88.9% exhibited regular shapes, 84.4% displayed well‐defined borders, 73.3% showed rich vascularity, and 59.1% featured a perilesional “vascular stalk sign.” Eighty percent of subungual glomus tumors presented with adjacent osseous involvement.

**Conclusion:**

The characteristic ultrasonographic presentation of subcutaneous glomus tumors comprises a hypoechoic mass with regular contours, distinct margins, and abundant internal vascular flow. Perilesional osseous changes can usually be observed in subungual glomus tumors.

## Graphical abstract

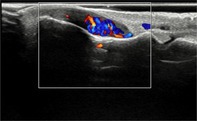

## 1 Introduction

Glomus tumors are benign vascular neoplasms arising from modified smooth muscle cells within the glomus body—a specialized thermoregulatory arteriovenous structure (Hufschmidt et al. 2017). These tumors account for 1%–2% of soft‐tissue tumors in the hand, with 75% demonstrating subungual localization (Mravic et al. 2015). The classic clinical presentation comprises intense pain (mean visual analog scale score: 7.0), point tenderness, and cold hypersensitivity, forming a diagnostic triad (Mortada et al. 2022). High‐frequency ultrasound (HFUS) serves as a well‐established, noninvasive imaging modality for superficial tissue evaluation (McCarthy and Thompson 2021; Yang et al. 2024), demonstrating high diagnostic sensitivity for glomus tumors through its ability to delineate characteristic features. Current literature predominantly documents these ultrasonographic findings in case reports (Saiyed et al. 2025; Wiese et al. 2024; Falcone et al. 2024). This retrospective cohort study quantitatively analyzes HFUS characteristics in 45 pathologically confirmed cases to establish evidence‐based imaging criteria and validate key diagnostic markers including the vascular stalk sign.

## 2 Materials and Methods

### 2.1 Study Cohort

Forty‐five patients with histologically confirmed glomus tumors were retrospectively analyzed after preoperative HFUS at Peking University Third Hospital from October 2019 to April 2025. The cohort comprised 29 females (64.4%) and 16 males (35.6%), aged 20–66 years (mean ± SD: 39.6 ± 13.1 years), with symptom duration ranging from 2 to 360 months (62.8 ± 68.1 months). Tumor locations included 25 subungual lesions, 5 in digital pulp, 10 in periarticular regions, and 5 at other sites. Clinical characteristics recorded included pain/tenderness (*n* = 35), paroxysmal pain (*n* = 28), cold‐induced exacerbation (*n* = 4), heat‐induced exacerbation (*n* = 1), and cutaneous hyperpigmentation (*n* = 25) (Table 1).

**TABLE 1:** Distribution of clinical characteristics of patients.

	Number of cases	Proportion
Gender		
Male	16	35.6%
Female	29	64.4%
Age (years)		
20–29	14	31.1%
30–39	13	28.9%
40–49	6	13.3%
50–59	8	17.8%
60–66	4	8.9%
Lesion location		
Hand	31	68.9%
Nail bed	24	53.3%
Digital pulp	5	11.1%
Thenar	2	4.4%
Forearm	1	2.2%
Elbow	3	6.7%
Upper arm	1	2.2%
Foot nail bed	1	2.2%
Knee	5	11.1%
Ankle	2	4.4%
Balanus	1	2.2%
Pain		
Yes	42	93.3%
No	3	6.7%
Skin color change		
Yes	25	55.6%
No	20	44.4%

### 2.2 Ultrasonographic Protocol

All examinations utilized a Samsung RS85 system with a LA4‐18B high‐frequency linear transducer (15–18 MHz). Image acquisition encompassed:

1. Morphometric analysis: Maximum tumor diameter on the largest transverse plane and skin‐to‐tumor distance measured perpendicular to the epidermis.
2. Qualitative assessment: Classification of echogenicity (hypoechoic, isoechoic, hyperechoic, heterogeneous), morphology (regular: oval/round; irregular), and margins (well defined/ill defined).
3. Vascular evaluation: Color Doppler and power Doppler were used with a pulse repetition frequency (PRF) of 0.8–1.2 kHz and optimized gain. Blood flow signals within the tumor were classified as rich (covering more than half of the tumor area), moderate (20%–50%), little (less than 20%), or absent, with additional documentation of flow distribution (peripheral/central/mixed) and the presence of the vascular stalk sign (dominant connecting vessel).

### 2.3 Statistical Analysis

Subungual versus extrasubungual comparisons used independent‐sample *t*‐tests. Statistical significance was defined at *p* < 0.05 using SPSS v26.0.

## 3 Results

The study cohort consisted of 45 glomus tumors confirmed by histopathology. Morphometric analysis demonstrated a mean tumor diameter of 0.7 ± 0.5 cm (range: 0.2–2.8 cm). Subungual tumors exhibited significantly smaller dimensions compared to extrasubungual tumors, with mean diameters of 0.6 ± 0.3 cm (0.2–1.4 cm) versus 1.0 ± 0.7 cm (0.3–2.8 cm), respectively (*p* = 0.020).

**FIGURE 1:**
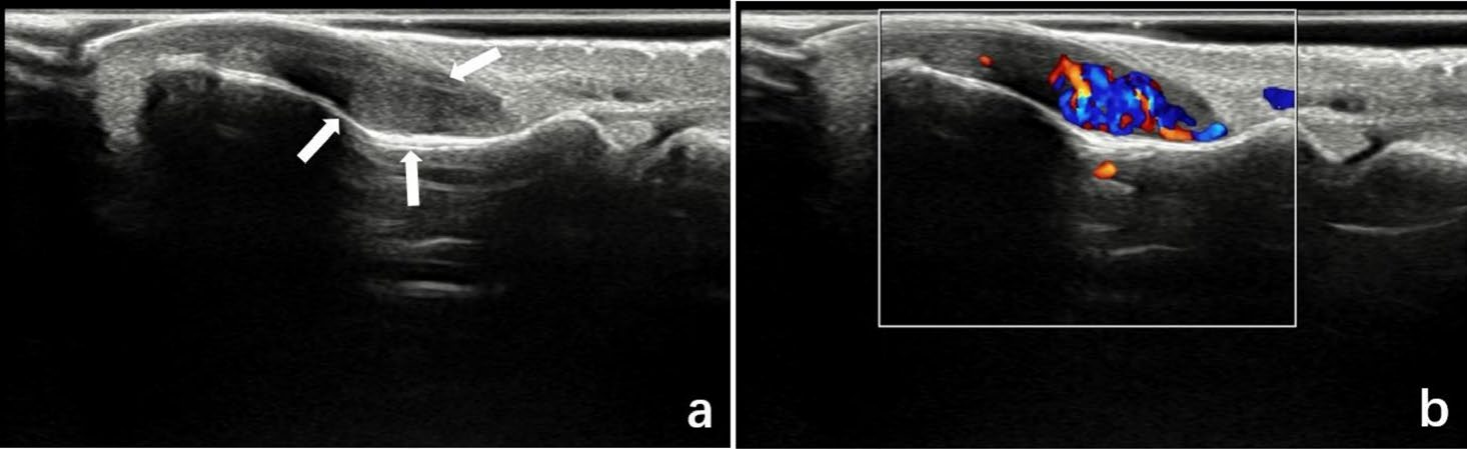
Subungual glomus tumor in a 37‐year‐old male (right thumb). (a) Grayscale ultrasound image demonstrates a well‐defined, ovoid hypoechoic mass (→) beneath the nail plate with posterior acoustic enhancement. (b) Color Doppler image reveals intralesional hypervascularity.

**FIGURE 2:**
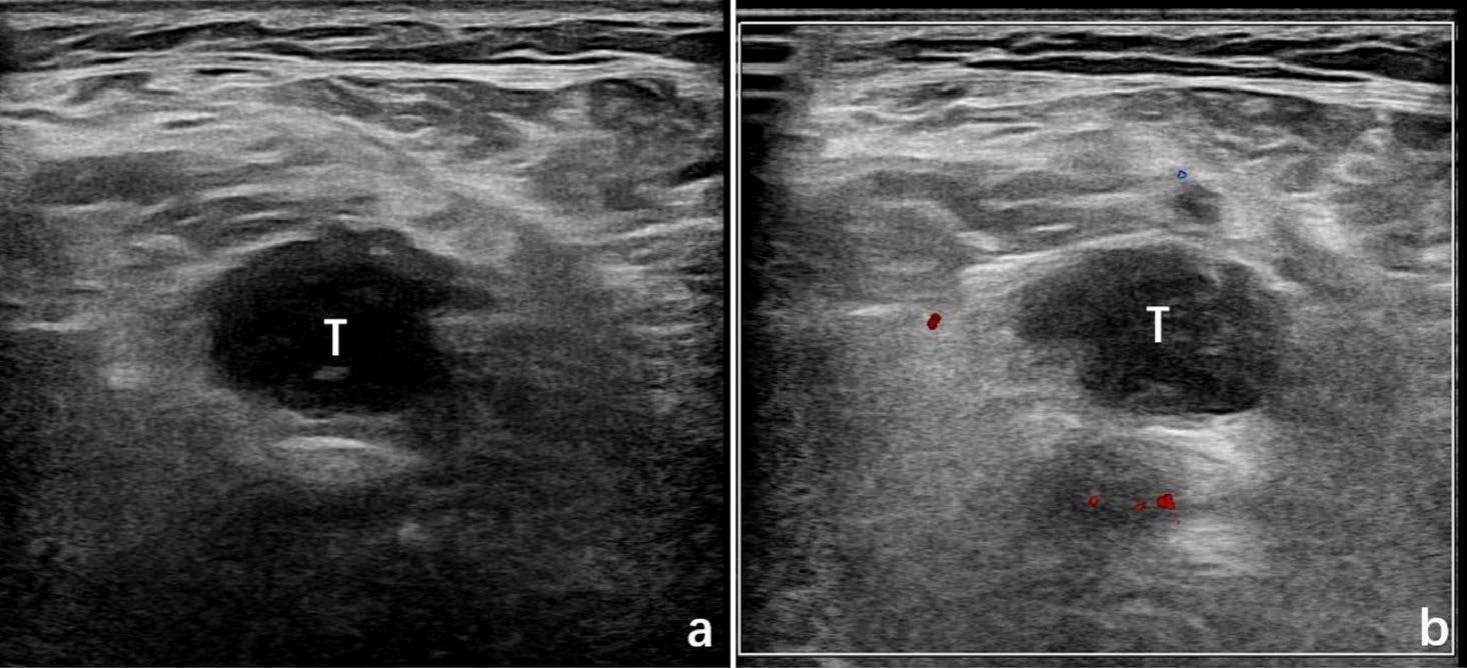
Extradigital glomus tumor in a 59‐year‐old male (left popliteal fossa). (a) Transverse grayscale ultrasound image shows an irregular hypoechoic lesion (T) within muscular tissue exhibiting posterior enhancement. (b) Absent vascularity on color Doppler.

**FIGURE 3:**
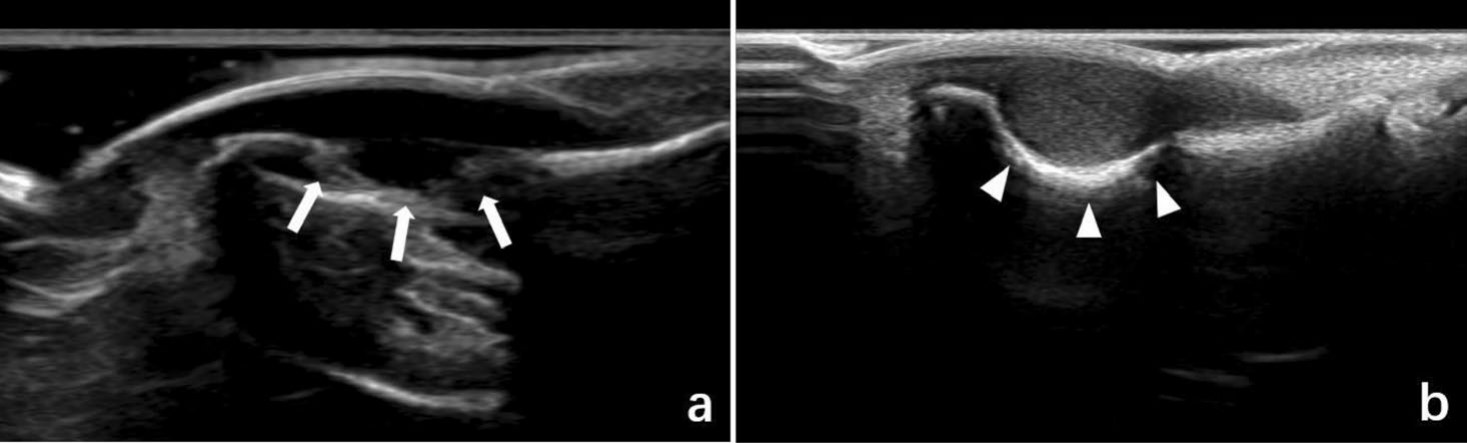
Osseous manifestations associated with subungual glomus tumors. (a) Cortical erosion (→) adjacent to tumor in a 30‐year‐old female (right fifth digit). (b) Pressure‐induced cortical deformation (△) in a 32‐year‐old female (right first digit).

**FIGURE 4:**
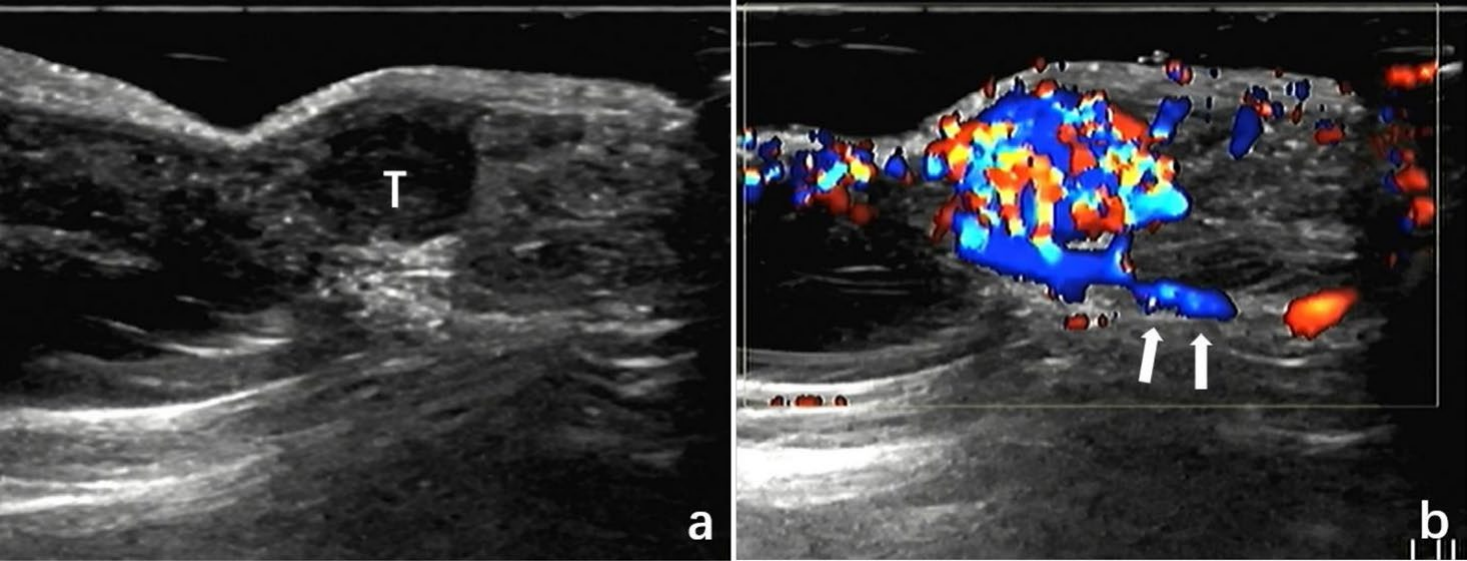
Extradigital glomus tumor in a 52‐year‐old female (left fourth digital pulp). (a) Gray‐scale ultrasound image shows that the tumor (T) is located within the digital pulp muscle. (b) Color Doppler shows intralesional hypervascularity and the presence of “vascular stalk sign” (→).

The shared sonographic characteristics of subungual and extrasubungual glomus tumors include hypoechoic echotexture, regular morphology, well‐defined margins, and rich vascularity. Adjacent osseous manifestations were observed in 44.4% (20/45) of all cases. Notably, all these osseous manifestations were observed in the subungual group, accounting for 80.0% (20/25) of subungual tumors. Comprehensive imaging characteristics are detailed in Tables 2, 3 and Figures 1, 2, 3, 4.

**TABLE 2:** Ultrasonic manifestations of the 45 glomus tumors.

Ultrasonic signs	Number of cases	Proportion
Echogenicity		
Hypoechoic	42	93.3%
Ioechoic	0	0.0%
Hyperechoic	0	0.0%
Heterogeneous	3	6.7%
Morphology		
Regular	40	88.9%
Round	10	22.2%
Oval	30	66.7%
Irregular	5	11.1%
Margins		
Well defined	38	84.4%
Ill defined	7	15.6%
Osseous manifestations		
Cortical erosion	5	11.1%
Cortical deformation	15	33.3%
Absent	25	55.6%
Calcification		
Present	0	0.0%
Absent	45	100.0%
Posterior enhancement		
Present	32	71.1%
Absent	13	28.9%
Vascularities		
Rich	33	73.3%
Moderate	8	17.8%
Little	3	6.7%
Absent	1	2.2%
Flow distribution		
Peripheral	14	31.8%
Central	6	13.6%
Mixed	24	54.5%
Vascular stalk sign		
Present	26	57.8%
Absent	19	42.2%

**TABLE 3:** Ultrasonic manifestations comparison of the 20 subungual and the 25 extrasubungual subcutaneous glomus tumors.

N (%)	Hypoechoic	Regular morphology	Well‐defined margins	Osseous manifestations	Rich vascularity	Vascular stalk sign
Subungual	24 (96.0%)	23 (92.0%)	18 (72.0%)	20 (80.0%)	18 (72.0%)	16 (64.0%)
Extrasubngual	18 (90.0%)	17 (85.0%)	20 (100.0%)	/	15 (75.0%)	10 (50.0%)

The depth from the tumors to the skin surface averaged 0.2 ± 0.3 cm (range: 0.1–1.8 cm). Notably, lesions presenting with cutaneous hyperpigmentation (*n* = 25; purple/red/purple) were significantly more superficial (mean depth 0.10 ± 0.03 cm) compared to tumors without skin changes (*n* = 20; mean depth 0.30 ± 0.40 cm), establishing a significant inverse relationship between the visibility of surface discoloration and tumor depth (*p* = 0.010).

## 4 Discussion

The demographic predominance of middle‐aged females in our cohort aligns with established epidemiological patterns of glomus tumors (Al‐Janabi et al. 2024; Nthumba and Oundoh 2024). Our patients exhibited a variable evolution period, ranging from months to decades. The diagnosis and treatment of glomus tumors are easily delayed because of their rarity (Chiang et al. 2014). Pain constitutes a hallmark symptom (Morey et al. 2016), though 6.7% (3/45) of cases presented asymptomatically—one subungual lesion and two extrasubungual tumors (thenar and upper arm). These atypical presentations—particularly extrasubungual lesions—increased the difficulty of diagnosis. This deviation from classical symptomatology underscores the diagnostic challenge for painless extrasubungual masses, necessitating multimodal imaging evaluation to mitigate false‐negative assessments (El Jouari et al. 2018). The depth‐dependent hyperpigmentation observed in superficial tumors likely stems from tumor‐induced angiogenesis and subsequent hemosiderin deposition. The skin color may not show any abnormalities when the tumor is deep (Gombos and Zhang 2008).

HFUS serves as a nonradiating and cost‐effective modality for characterizing these tumors, particularly given their predominantly superficial location. Key sonographic features of subcutaneous glomus tumors include hypoechoic echotexture, regular morphology, well‐defined margins, rich vascularity, the vascular stalk sign, and osseous involvement. These sonographic findings of glomus tumors are consistent with previous reports (Lee et al. 2015; Van Houtven et al. 2024; Jamalipour Soufi et al. 2024). In our study, only 84.4% of tumors showed well‐defined margins. Image resolution improves as ultrasound frequency increases. Our study has certain technical limitations that warrant acknowledgment. First, regarding the ultrasound frequency, the 15–18 MHz linear transducer used in this study, while effective for visualizing most superficial glomus tumors, is not considered an ultrahigh‐frequency transducer for skin‐focused ultrasound. Higher‐frequency probes, for example, 33 MHz, which are more commonly utilized in dermatological ultrasound, can delineate finer structural details such as submillimeter tumor margins (Ávila de Almeida et al. 2021).

The vascular stalk sign's 59.1% prevalence provides a specificity marker (Chen et al. 2020; Park et al. 2011), attributable to perforating vessel recruitment during tumor growth. Although color and power Doppler successfully identified rich intratumoral vascularity in 73.3% of cases, these conventional modes are less sensitive compared to the microvascular imaging systems integrated into the Samsung RS85 scanner employed in this study. Superb microvascular imaging (SMI) enables the evaluation of low‐velocity blood flow without contrast agents, which makes it safer and more cost‐effective than contrast‐enhanced ultrasound. Additionally, SMI performs better than color and power Doppler imaging in detecting and characterizing microvessels (Jiang et al. 2019; Tang et al. 2022; Corvino et al. 2022). However, due to the retrospective nature of this study and the lack of standardized microvascular imaging protocols at the time of data collection, only color and power Doppler were used for vascular assessment in the included cases. This limitation may have resulted in underreporting of subtle vascular patterns, such as sparse peripheral microvessels, in some small or minimally vascularized glomus tumors, potentially affecting the comprehensiveness of our vascular characterization.

Osseous involvement comprised cortical erosion and pressure‐induced deformation, reflecting mechanical displacement within confined subungual spaces (Baek et al. 2010). Cortical erosion caused by glomus tumors is rare, with only a few case reports documented (Bouayyad et al. 2020; Abidin et al. 2023; Solunke et al. 2024). Pressure‐induced deformations caused by glomus tumors have been reported in a study of 50 cases (Fan et al. 2016). These pressure‐induced deformations exhibited trabecular remodeling without osteolysis, suggesting mechanical adaptation rather than invasive growth. Osseous involvement of subungual glomus tumors was common in this study and represented a typical ultrasonic manifestation.

Contrary to Takanashi et al.'s report of smaller extrasubungual tumors (Takanashi et al. 2023), our data indicate larger dimensions in extrasubungual versus subungual lesions (1.0 ± 0.7 vs. 0.6 ± 0.3 cm; *p* = 0.020). The dimensional discrepancy between subungual and extrasubungual tumors may reflect anatomical constraints: the rigid nail matrix likely restricts lateral expansion, whereas softer tissues permit volumetric growth. However, both studies are constrained by limited sample sizes, precluding definitive morphological generalizations.

A limitation of this study is that we did not utilize higher‐frequency probes or SMI to evaluate glomus tumors. Notably, this study represents one of the largest single‐center cohorts for glomus tumors—a rare disease—comprising 45 pathologically confirmed cases. This sample size enabled subgroup analyses (subungual vs. extrasubungual glomus tumors) and systematic characterization of their ultrasonographic features, providing more robust evidence for clinical practice than single‐case reports. Future studies could address this limitation by adopting ultrahigh‐frequency probes for imaging and incorporating microvascular imaging techniques to capture more detailed structural and vascular information of glomus tumors. Additionally, the single‐center design may introduce selection bias, particularly for extrasubungual cases. Future multicenter studies could validate our sonographic criteria using standardized imaging protocols.

## 5 Conclusion

HFUS reliably identified characteristic ultrasonographic features of subcutaneous glomus tumors, including hypoechoic morphology, well‐defined margins, and Adler grade III vascularity. The vascular stalk sign provides additional diagnostic specificity by demonstrating vascular continuity with adjacent tissues. Additionally, osseous changes can serve as a typical ultrasonic manifestation of subungual glomus tumors. HFUS also enables the detection of small lesions and atypical painless cases, confirming its utility in preoperative localization and differential diagnosis.

## Funding

The authors received no specific funding for this study.

## Ethics Statement

The study was approved by the Ethics Committee of Peking University Third Hospital in 2025 (ethical approval number: 2025‐5960‐01, date of approval: August 7, 2025).

## Conflicts of Interest

The authors declare no conflicts of interest.

## Data Availability

The data that support the findings of this study are available from the corresponding author upon reasonable request.
